# Expression of ABC Efflux Transporters in Placenta from Women with Insulin-Managed Diabetes

**DOI:** 10.1371/journal.pone.0035027

**Published:** 2012-04-27

**Authors:** Gregory J. Anger, Alex M. Cressman, Micheline Piquette-Miller

**Affiliations:** 1 Department of Pharmaceutical Sciences, Leslie Dan Faculty of Pharmacy, University of Toronto, Toronto, Ontario, Canada; 2 Department of Pharmacology and Toxicology, Faculty of Medicine, University of Toronto, Toronto, Ontario, Canada; University of Cambridge, United Kingdom

## Abstract

Drug efflux transporters in the placenta can significantly influence the materno-fetal transfer of a diverse array of drugs and other xenobiotics. To determine if clinically important drug efflux transporter expression is altered in pregnancies complicated by gestational diabetes mellitus (GDM-I) or type 1 diabetes mellitus (T1DM-I), we compared the expression of multidrug resistance protein 1 (MDR1), multidrug resistance-associated protein 2 (MRP2) and the breast cancer resistance protein (BCRP) via western blotting and quantitative real-time polymerase chain reaction in samples obtained from insulin-managed diabetic pregnancies to healthy term-matched controls. At the level of mRNA, we found significantly increased expression of MDR1 in the GDM-I group compared to both the T1DM-I (p<0.01) and control groups (p<0.05). Significant changes in the placental protein expression of MDR1, MRP2, and BCRP were not detected (p>0.05). Interestingly, there was a significant, positive correlation observed between plasma hemoglobin A1c levels (a retrospective marker of glycemic control) and both BCRP protein expression (r = 0.45, p<0.05) and BCRP mRNA expression (r = 0.58, p<0.01) in the insulin-managed DM groups. Collectively, the data suggest that the expression of placental efflux transporters is not altered in pregnancies complicated by diabetes when hyperglycemia is managed; however, given the relationship between BCRP expression and plasma hemoglobin A1c levels it is plausible that their expression could change in poorly managed diabetes.

## Introduction

Over 220 million people worldwide are diagnosed with diabetes and rising prevalence is reported in nearly all surveyed populations [1], [2]. Accordingly, the percentage of pregnancies affected by pre-existing type 1 (T1DM) and type 2 diabetes mellitus (T2DM) or by diabetes that develops during the second and third trimesters of pregnancy, called gestational diabetes mellitus (GDM), is also on the rise. In the context of pharmacokinetic variation, it is known that diabetes alters drug-disposition mechanisms in non-pregnant subjects, as reviewed by Gwilt and colleagues [3], but the impact of diabetes on drug disposition in pregnancy has not been thoroughly evaluated. Atypical drug disposition in pregnancy has implications for both maternal and fetal health, as the effectiveness of clinically important therapeutics may be compromised.

**Figure 1:**
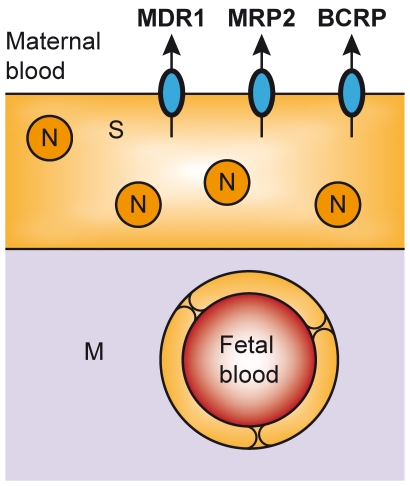
Localization of MDR1, MRP2, and BCRP in placenta. The syncytiotrophoblast (S) layer of the placenta is comprised of multinucleated (N) cells that contain a number of apically-localized transport proteins that contribute to the function of the placental barrier, including MDR1, MRP2, and BCRP.

Drugs, xenobiotics, and their metabolites encounter a variety of epithelial and endothelial barriers as they make their way to their site(s) of action and elimination. To circumvent these barriers, they are required to cross cell membranes. Multidrug resistance protein (MDR) 1 (P-glycoprotein; Gene: ABCB1), multidrug resistance-associated protein 2 (MRP2; Gene: ABCC2) and the breast cancer resistance protein (BCRP; Gene: ABCG2) are abundantly expressed ATP-binding cassette (ABC) drug efflux transporters found in the apical localization of the placental syncytiotrophoblast layer (Fig. 1). Here they limit the materno-fetal transfer of a diverse array of drug substrates by the ATP-driven efflux of substrates from the fetal compartment into the maternal circulation [4], [5], [6]. The importance of these transporters in determining fetal xenobiotic exposure has been well-established in placental cell lines and placental primary cultures [7], in mutant/knockout mice [8], [9], [10], [11], and in *ex vivo* placental perfusion models [12], [13], [14]. In one such study, relative to controls, pregnant Mdr1 gene knockout mice (Mdr1^-/-^ mice) treated with the Mdr1/Bcrp-specific inhibitor GF120918 exhibited twice the fetal penetration of the Mdr1/Bcrp substrate topotecan, a DNA topoisomerase inhibitor and potent antineoplastic agent [6]. Whereas lack of or decreased expression can lead to increased exposure to xenobiotics, over expression may result in decreased exposure. This may result in clinical consequences when treating fetal illness (e.g. fetal arrhythmias with digoxin, a MDR1 substrate [15]) or by limiting the maternal-fetal passage of therapeutics believed to contribute to the prevention of vertical disease transmission (e.g. highly active antiretroviral therapy in antenatal HIV infection).

We have previously demonstrated that placental MDR1, MRP2 and BCRP mRNA expression is elevated in rat dams with streptozotocin-induced gestational diabetes mellitus (GDM) but nearly normal in rat dams with streptozotocin-induced GDM whose blood glucose was normalized following insulin treatment [16]. In the aforementioned study, relative to controls, fetal exposure to the MDR1 substrate lopinavir, a commonly used HIV protease inhibitor, was found to be dramatically lower in rat dams with streptozotocin-induced GDM [16]. These profound results and the increasing prevalence of diabetes mellitus in pregnancy prompted us to study the impact of GDM and T1DM on placental transporter expression.

Therefore, in the present study, we examined mRNA and protein expression of MDR1, MRP2 and BCRP in placentas from pregnancies complicated by type 1 diabetes mellitus (T1DM-I) or gestational diabetes mellitus (GDM-I). All available placental samples were obtained from patients whose glycemia was managed with insulin treatment. We hypothesized that placental drug efflux transporter expression may be altered in individuals who have diabetes, but could be normalized to levels found in healthy term controls in patients with well-managed diabetes based on our previous preclinical findings in pregnant animal models of non-treated and insulin-treated diabetes mellitus [16].

## Methods

### Sample Acqu1isition

Thirty-five human specimens from pregnancies meeting our inclusion/exclusion criteria (Table 1) were obtained by the Research Centre for Women’s and Infants’ Health (RCWIH) BioBank program of Mount Sinai Hospital, in accordance with the policies of the Mount Sinai Hospital Research Ethics Board. Sample acquisition and aspects of placental processing and measurement are extensively described on the RCWIH BioBank’s website: http://biobank.lunenfeld.ca. Briefly, immediately after delivery, large tissue cores through the full thickness of the placenta were obtained in each quadrant, avoiding chorionic plate tissue and areas with obvious evidence of thrombosis or other abnormalities. Samples were snap-frozen in liquid nitrogen and stored at −80°C until use. All clinical data that was extracted from patient charts and collected by the RCWIH BioBank program of Mount Sinai Hospital was made available to this study. In accordance with the recommendations of Nelson and Burton [17], supplemental tables describing key patient variables were compiled (See data S1). Extensive chart review was completed to ensure that all patients met our inclusion/exclusion criteria and that any medications patients were taking were recorded. Samples included in this study reflect total RCWIH BioBank availability between September 2006 and February 2012: 8 T1DM-I samples, 13 GDM-I samples, and 14 randomly selected term controls.

**Table 1:** Inclusion/exclusion criteria.

Group	Inclusion criteria	Exclusion criteria
Term control	• >37 weeks + 0 days	• Pertaining to diabetes status:o T1DM, T2DM, or GDM• Other:o Hypothyroidism or hyperthyroidismo Cushing’s diseaseo Essential hypertensiono Pregnancy-induced hypertensiono Arrhythmiaso Rheumatic fevero Thalassemiao Sickle cell anemiao von Willebrand diseaseo Immunothrombocytopeniao Multiple sclerosiso Epilepsyo Bipolar (medicated)o Systemic lupus erythematosuso Antiphospholipid antibody syndromeo Rheumatoid arthritiso Inflammatory bowel diseaseo Irritable bowel syndromeo Chorioamnionitiso Group B streptococcuso Sexually transmitted infectionso Urinary tract infectionso H1N1 during pregnancyo Gallstones or kidney stoneso Renal or liver diseaseo Cholestasis of pregnancyo Placenta increta/percretao Placental abruptiono Pelvic inflammatory diseaseo Recovered from cancero Smoking during pregnancy
T1DM-I	• T1DM (insulin-managed)• >37 weeks + 0 days	• Pertaining to diabetes status:o T2DM• Other:o Same as term control group
GDM-I	• GDM (insulin-managed)• >37 weeks + 0 days	• Pertaining to diabetes status:o T1DM, T2DM, GDM (diet-managed)• Other:o Same as term control group

### Hemoglobin A1c Level Determination

HbA1c levels were determined by GLP certified laboratories using the Roche Cobas Integra Clinical Chemistry System. In this assay, all hemoglobin variants which are glycated at the β-chain N-terminus and which have antibody-recognizable regions identical to that of HbA1c are determined. HbA1c is presented as a percentage of total hemoglobin (% HbA1c).

### RNA Extraction and cDNA Synthesis

Total RNA was extracted from 60 mg of placental tissue using the TRIzol method (Invitrogen, Carlsbad, CA) and subjected to qualitative and quantitative measurements (A_260/280_>1.90 and A_260/320_>1.60) using a NanoDrop spectrophotometer (Thermo Fisher Scientific, Waltham, MA). 4 µg of total extracted RNA was reverse transcribed to cDNA using the Maxima First Strand cDNA Synthesis Kit for RT-qPCR (MBI Fermentas, Hanover, MD), all according to the manufacturer’s instructions. Briefly, 4 µL of reaction mix (containing reaction buffer, dNTPs, oligo and random hexamer primers), 2 µL of Maxima Enzyme mix containing Maxima Reverse Transcriptase and RiboLock RNase inhibitor, sample RNA, and nuclease-free H_2_O were added and centrifuged. Samples were then reverse-transcribed in an Eppendorf Thermocycler (Eppendorf AG, Hamburg, Germany) with the following thermocycling parameters: 10 min at 25°C, 30 min at 50°C, and 5 min at 85°C.

### Real-Time Quantitative Polymerase Chain Reaction (qPCR)

MDR1 (ABCB1), MRP2 (ABCC2), and BCRP (ABCG2) mRNA expression levels in placentae from T1DM-I, GDM-I, and term-matched controls were determined by real-time quantitative reverse-transcriptase polymerase chain reaction. RT-qPCR was performed on cDNA using LightCycler technology with SYBR Green I fluorescence detection (Roche Diagnostics, Montreal, QC). PCR oligonucleotides were synthesized at The Hospital for Sick Children (DNA Synthesis Centre, Toronto, ON) and were designed to span adjacent exon-exon junctions to eliminate the amplification of genomic DNA. Primer sequences were as follow: 5′-ACCAGATAAAAGAGAGGTGCAACGG-3′ (MDR1-F), 5′-TCCCGGCCCGGATTGACTGA-3′ (MDR1-R), 5′-AGCAGCCATAGAGCTGGCCCTT-3′ (MRP2-F), 5′-AGCAAAACCAGGAGCCATGTGCC-3′ (MRP2-R), 5′-AGCAGCTCTTCGGCTTGCAACA-3′ (BCRP-F), 5′-GTTCCAACCTTGGAGTCTGCCACT-3′ (BCRP-R), 5′-AGCCTCGCCTTTGCCGATCC-3′ (β-actin-F), and 5′-TTGCACATGCCGGAGCCGTT-3′ (β-actin-R). Briefly, 2 µL of cDNA was added to 18 µL of reaction mix containing 4 µL of Roche SYBR green, 100pmol of forward and reverse qPCR primers, and nuclease-free H_2_O. Sequences were amplified using the following thermocycling parameters: 1 cycle of 10 min at 95°C, 50 cycles (6 seconds at 95°C, 5 seconds at 55°C, and 6 seconds at 72°C), followed by a continuous 150-second melt curve from 65–99°C. A melt-curve was used after amplification to ensure primer specificity such that all samples exhibited a single amplicon. No template control samples were also run to confirm that samples and primers were not contaminated with any genomic DNA.

**Figure 2:**
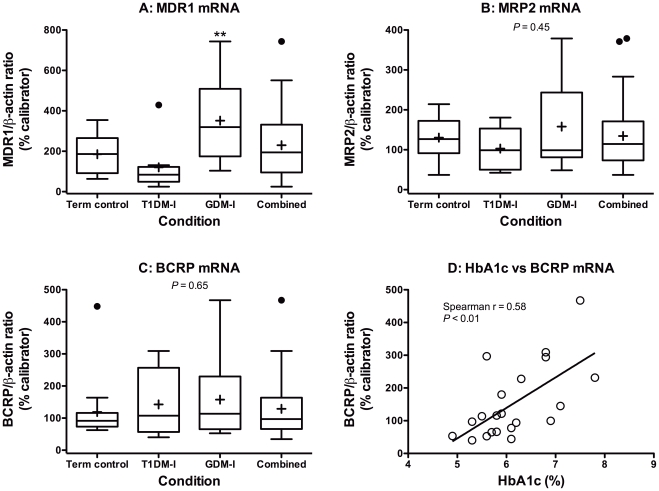
Placental ABC efflux transporter expression in pregnancies complicated by insulin-managed diabetes. **A**, MDR1 (ABCB1). **B**, MRP2 (ABCC2). **C**, BCRP (ABCG2). Boxes represent quartiles enclosing 50% of the data and the whiskers at either end extend to the 25^th^ and 75^th^ quartiles. The median is marked by a line, the mean is marked by an “+” and outliers are marked by a “•” outside the 25^th^ and 75^th^ quartiles. The “Combined” group presents data from all patients (n = 33) and was not included in statistical analyses. **D**, Spearman correlation analysis of the relationship between HbA1c levels (%) and BCRP mRNA expression in diabetic pregnancies (p<0.01).

Expression levels of transporters and β-actin mRNA as C_q_ (the point at which gene of interest and reference gene could be quantified) were quantified using the Roche LightCycler II software (Ver. 3.5) configured with the Roche LightCycler II real-time qPCR instrument (Roche Diagnostics GmbH, Hamburg, Germany). Gene-of-interest expression was normalized to β-actin (ACTB) mRNA expression, using the efficiency-corrected ΔC_t_ method and presented as a gene-to-β-actin ratio. The efficiency-corrected ΔC_t_ method was used because there were slight differences in the efficiencies of our primer sets as determined by standard curve analysis of serially-diluted calibrator cDNA. Gene-to-β-actin ratios were then converted to percentages of a calibrator sample and presented as such in figure 2.

### Western Blotting

300 mg of placental samples were homogenized using a motorized pestle in radioimmunoprecipitation assay (RIPA) buffer, containing freshly added dithiothrietol (1 mM; DTT; Sigma-Aldrich, Oakville, ON), phenylmethylsulfonyl fluoride (0.5 mM; PMSF; BioShop Canada Inc., Burlington, ON) and 1X protease inhibitor cocktail (Sigma-Aldrich, Oakville, ON). Homogenates were then incubated on ice for 30 min with brief vortex mixing at 15 min and subsequently centrifuged at 18,000 *g* for 15 min. For each sample, the supernatant was isolated and subjected to a Bradford assay [18]. Samples containing 60 µg of protein in Laemmli sample loading buffer were heated at 36°C for 20 min and then separated via 8% sodium dodecyl sulfate-polyacrylamide gel electrophoresis and transferred to polyvinylidene difluoride (PVDF) membranes (Bio-Rad Laboratories Canada, Ltd., Mississauga, ON). PVDF membranes were blocked in 5% non-fat dry milk in TBST and incubated with an anti-MDR1 mouse monoclonal antibody (C219 clone, 1∶500, ID Labs Biotechnology, Inc., London, ON), anti-MRP2 mouse monoclonal antibody (M2 III-6 clone, 1∶100, Abcam, Inc., Cambridge, MA) or anti-BCRP rat monoclonal antibody (BXP-53 clone, 1∶50, Abcam, Inc., Cambridge, MA) in 2% non-fat milk TBST. These MDR1, MRP2, and BCRP antibodies are commercially available and produce distinct, quantifiable bands when used as described in the next paragraph (data S2). Furthermore, they have previously been utilized to analyze the expression of transporters in both human and animal tissues [19], [20], [21].

After a series of washes with TBST, membranes were incubated with a horseradish peroxidase-labeled secondary antibody (Jackson ImmunoResearch Laboratories, Inc., West Grove, PA) in 2% non-fat milk TBST. Following a series of washes with TBST, immunoreactive proteins were detected in membranes using ECL Plus chemiluminescence (Amersham Biosciences, Baie d’Urfé, QC). The optical density (OD) of each band was determined using ImageJ for Macintosh (version 1.44o) software. To confirm equivalent protein loading, immunodetection using an anti-β-actin monoclonal antibody was employed (AC-15, 1∶30,000, Sigma-Aldrich, Oakville, ON) so that the OD of MDR1, MRP2 and BCRP could be normalized internally to β-actin. To correct for gel-to-gel variability, a calibrator sample was run in each gel. Protein-of-interest/β-actin ratios for each blot were converted to percentages of that blot’s calibrator sample’s ratio.

### Statistics

Patient and transporter expression data were analyzed using GraphPad Prism 5.0 for Macintosh (GraphPad Software, Inc., San Diego, CA). Patient clinical characteristics were all analyzed using the ANOVA followed by the post hoc Bonferroni’s multiple comparison test to assess for subgroup differences. Western blot and quantitative real-time PCR results are reported as protein (or gene)-of-interest/β-actin ratio as % calibrator for each placental group. Protein (or gene)-of-interest/β-actin ratio as % calibrators were compared by ANOVA followed by the post hoc Dunn’s or Bonferroni’s multiple comparison test to assess for subgroup changes in protein and mRNA expression. Spearman coefficient analysis was used to determine the correlation between the levels of mRNA and protein expression of MDR1, MRP2, and BCRP transporters to HbA1c levels. Power analysis was performed using G*Power 3.1 for Macintosh [22]. Levels of significance for statistical analyses were set at or below α = 0.05, indicated as follows: */#, p<0.05 and **/##, p<0.01. All results are presented as mean±S.D.

## Results and Discussion

There are a number of short- and long-term maternal and fetal consequences of unmanaged, or poorly managed, diabetes in pregnancy. Throughout gestation, pregnant women with diabetes can experience polyuria, polydipsia and hyperphagia (the classical triad of diabetes symptoms). Pregnant women with diabetes can also experience hyperlipidemia [23], [24], [25], [26] and reduced weight gain [27]. For the fetus, in the prenatal period, there is increased production of fetal insulin, which leads to increased storage of excess glucose as fetal adipose tissue and the development of macrosomia [28], [29]. Placental enlargement is also a feature of pregnancies affected by diabetes [30], [31], [32], [33], [34], [35], [36]. With T1DM, shorter gestational periods have been reported [33]. The long-term consequences of unmanaged, or poorly managed, diabetes in pregnancy revolve primarily around the risk of future metabolic disease in both mother, when GDM, and child [28], [29], [37], [38]. These consequences, both short- and long-term, are believed to be directly related to the degree of maternal hyperglycemia (i.e., the severity of maternal disease across gestation).

Patient data for the groups with diabetes indicate that only the T1DM-I group displayed characteristics of poorly managed diabetic pregnancy (Table 2; data S1). In the T1DM-I group, gestation was significantly shorter (p<0.01) and there was a trend toward larger placental volume (p = 0.07). Despite T1DM mothers having a shorter gestational age, their neonates actually trended towards a higher birth weight (Table 2). It is possible that neonates in this condition would have become significantly heavier had their gestations been of equivalent duration. Nonetheless, the absence of significant differences in neonatal birth weight, particularly given the strong correlation that exists between neonatal birth weight and maternal blood glucose concentrations, could signify adequate glycemic control in this study’s T1DM-I and GDM-I subgroups [39], [40].

**Table 2:** Clinical data.

Characteristic	Control (n = 14)	T1DM (n = 8)	GDM (n = 13)
Age (years)	32±4	33±3	35±4
Ethnicity			
Caucasian	11 (79)	6 (75)	7 (54)
Asian	3 (21)	0 (0)	1 (8)
Black	0 (0)	0 (0)	2 (15)
Other	0 (0)	2 (25)	3 (23)
BMI	23.2±4.5	25.9±3.9	29±6.6*
Duration of diabetes (years)	N/A	17±9	<1
HbA1c (%)^a^	N/A	6.4±0.6	6±0.8
Therapeutics used^b^ (#)	2±1	4±2	3±1
Gravidity (#)	2.3±1.1	3.2±2.2	2.2±0.9
Parity (#)	1.8±0.8	2.1±1.2	1.8±0.7
Gravidity/parity ratio	1.3±0.6	1.6±0.4	1.2±0.3
GBS status			
Positive	0 (0)	0 (0)	0 (0)
Negative	14 (100)	8 (100)	8 (69)
Unknown	0 (0)	0 (0)	4 (31)
Mode of delivery			
Vaginal	5 (36)	4 (50)	2 (15)
C-section	9 (64)	4 (50)	11 (85)
Rupture of membranes			
SROM	3 (21)	2 (25)	1 (8)
AROM	11 (79)	6 (75)	12 (92)
Gestational age (days)	275±7	266±6**	271±5
Neonatal birth weight (g)	3481±417	3705±542	3301±404
Neonatal sex			
Male	7 (50)	6 (75)	5 (38)
Female	7 (50)	2 (25)	8 (62)
Placental weight^c^ (g)	608±127	666±101	661±175
Placental volume^d^ (cm^3^)	681±174	977±381	766±283
Neonate:placenta weight ratio	5.9±0.8	5.4±0.9	5.2±1.1

Statistical results are presented as mean±SD. All other results are presented as [n (%)].

* , Significantly different from term controls (*, p<0.05; **, p<0.01). N/A, not applicable or not available.

a HbA1c values were measured in the 2^nd^ and/or 3^rd^ trimester and are presented as group means, based on 1–5 individual measurements.

b Includes drugs, vaccines and vitamin supplements.

c Wet, untrimmed weight.

Following a high fasting blood glucose reading or high oral glucose tolerance test, women are diagnosed with diabetes and their HbA1c levels monitored over time to track glyemic control. In proportion to blood concentrations, glucose is able to react with normal hemoglobin (HbA0) in red blood cells to produce HbA1c. HbA1c concentrations, therefore, reflect average blood glucose concentrations over the previous 3–4 months, which is the approximate lifespan of red blood cells [41]. Murphy and colleagues found that maintaining lower HbA1c levels in the third trimester of pregnancy by way of continuous blood glucose monitoring was associated with decreasing risk of fetal macrosomia [42], which is thought to be the result of prolonged periods of glucose mismanagement. Mean HbA1c values for both diabetic groups fell within acceptable ranges for managed diabetes (i.e., ≤6.5% HbA1c), which is again supportive of proper glycemic control at the group level. At the individual level, though, 4 T1DM-I group members and 2 GDM-I group members exhibited HbA1c levels that exceeded 6.5%, which indicates some degree of mismanagement within the DM groups. Clinical data for a measure of glycemic control, glycosylated hemoglobin (HbA1c), was available only for the diabetic groups due to the inappropriateness of routinely conducting this test in healthy term-control populations.

**Figure 3:**
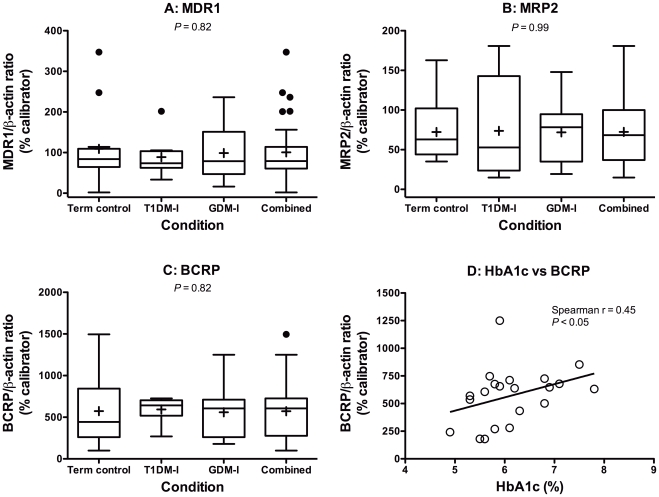
Placental ABC efflux transporter expression in pregnancies complicated by insulin-managed diabetes. **A**, MDR1 (ABCB1). **B**, MRP2 (ABCC2). **C**, BCRP (ABCG2). Boxes represent quartiles enclosing 50% of the data and the whiskers at either end extend to the 25^th^ and 75^th^ quartiles. The median is marked by a line, the mean is marked by an “+” and outliers are marked by a “•” outside the 25^th^ and 75^th^ quartiles. The “Combined” group presents data from all patients (n = 33) and was not included in statistical analyses. **D**, Spearman correlation analysis of the relationship between HbA1c levels (%) and BCRP protein expression in diabetic pregnancies (p<0.05).

Very little attention has been paid to drug disposition mechanisms, such as placental transporter expression and/or function, in pregnancies affected by T1DM or GDM. In this study, average placental MRP2 and BCRP mRNA and protein expression in the T1DM-I and GDM-I groups was not statistically different from controls (Fig. 2B, 3B, 2C, 3C). Placental MDR1 mRNA expression was significantly higher in the GDM-I group, relative to controls, but this did not translate into significantly higher MDR1 protein expression (Fig. 2A and 3A). Normal expression in these insulin-managed groups, with the exception of MDR1 mRNA in GDM-I, is consistent with our observations in insulin-managed rat dams with streptozotocin-induced GDM [16].

Interestingly, when examining individual rather than mean levels, plasma HbA1c levels were found to positively correlate with BCRP mRNA (r = 0.58; p<0.01; Fig. 2D) and protein (r = 0.45; p<0.05; Fig. 3D) expression in both insulin-managed diabetes groups. Significant correlations were not seen with MDR1 and MRP2 mRNA or protein expression. The strong relationship between glycemic control and BCRP expression in pregnancies complicated by insulin-managed diabetes may indicate that placental BCRP is particularly responsive to periods of hyperglycemia. Future mechanistic studies will follow up on this hypothesis. BCRP’s substrates are known to include many glucuronide and sulfate conjugates, antineoplastics, antibiotics, antiretrovirals, oral hypoglycemic agents, statins, natural health products, and several dietary carcinogens [43], [44], [45], [46], [47], [48].

The concept of glucose mismanagement during insulin-managed GDM or T1DM pregnancies has not been well investigated in the literature. To treat diabetes mellitus in pregnancy, exogenous insulin injections are typically self-administered and supplementary insulin tables are used to adjust dosages to meet specific blood glucose goals of individual patients across gestation. Given that many of the women in our study were experiencing diabetes for the first time (i.e. GDM) and that glucose levels and demands can change rapidly over gestation, the regular administration of parenteral insulin in combination may have resulted in patient failures in management of hyperglycemia. Clinical statistics for diabetes mismanagement are limited in pregnancy; however, reports in the literature illustrate that the percentage of patients with GDM who do not achieve their specific blood glucose goals falls between 20–30% [49], [50]. In the context of our study, we observed 6 of the 21 women in the combined diabetic groups with HbA1c levels that exceeded 6.5%. This equates to glucose mismanagement of approximately 28% in our clinical population, which is in agreement with the range proposed from Langer’s more powerful studies. Unfortunately, our study lacked statistical power to determine whether women with higher HbA1c levels, who are presumed to have had poorer glycemic control, also had statistically higher levels of BCRP mRNA and protein.

Using animal model-derived effect sizes of 0.78 for Mdr1 (200% mRNA increase, CV = 42%), 0.53 for Mrp2 (456% mRNA increase, CV = 120%) and 0.67 for Bcrp (230% mRNA increase, CV = 53%), a total sample size of 39 patients (13/group) would be required to achieve power ≥ 0.8 in an F test for all of the transporters examined in this study. The actual power achieved for MDR1, MRP2 and BCRP analyses in this study, based on these animal model-derived effect sizes, was 0.98, 0.77 and 0.93, respectively (group sizes were averaged for these post hoc power calculations); however, clinical effect sizes are often smaller than effect sizes in animal models. Because placental transporter expression data from unmanaged, or poorly managed, diabetes is unavailable, the true effect size is currently unknown.

In addition to exhibiting smaller effect sizes, clinical data is typically more variable than data obtained in animal models. The overall coefficient of variation, a measure of variation about the mean, for MDR1, MRP2 and BCRP protein expression in this study was found to be 71%, 61% and 54%, respectively. This is consistent with our animal model data [16] and comparable to other studies of transporter expression in human term placenta [20], [51]. Interestingly, interindividual transporter expression variation, as demonstrated in this study, appears to be small in comparison to other tissues. For example, 55-fold, 20.5-fold and 20-fold variation in human hepatic MDR1 has been observed [52], [53], [54]. Similarly, there are reports of 6-fold and 3-fold variation in human intestinal MDR1 [55], [56].

The strengths of our study lie in the novelty of our findings given that placental transporter expression in GDM-I and T1DM-I has yet to be investigated in a clinically relevant population. Also, our retrospective design allowed for “real-world” results allowing for the possibility of patient non-adherence in their insulin dosage regimens. It is highly plausible that in a true prospective research design where clinical data were collected over time, we may not have observed any such effect due to the ethical requirements of medical staff to recommend changes in insulin dosing or to administer a therapeutic alternative to insulin (e.g., oral hyopoglycemics) to help better manage blood glucose in non-adherent patients.

Despite the strength of our retrospective BioBank design, there were some limitations to our work. Firstly, the RCWIH BioBank was only able to extract information contained within the patient charts of donors to the BioBank program. This resulted in a lack of all possible clinical data (e.g., lipid profiling) that could have shed additional light on our patient populations. Also, despite observing significant correlations in our groups with the number of samples that were available, additional samples could have been added to further increase the power and magnitude of our study’s findings. Lastly, the fact that HbA1c levels were not available for our term-matched control patients is a limitation of our study but, clinically, an HbA1c assay is not typically ordered for patients with no history of glucose mismanagement. Samples from pregnancies with documented mismanagement of blood glucose were unavailable.

In conclusion, there are two particularly important aspects of this study. First, it suggests that placental transporter expression in well-managed T1DM-I and a GDM-I pregnancy is not significantly different from healthy term controls. However, this may not hold true when hyperglycemia is unmanaged. Supporting this is the observed significant correlation between HbA1c and BCRP mRNA and protein in both insulin-managed diabetes groups. Increased levels of HbA1c were associated with increased levels of BCRP mRNA and protein and could have implications for maternal-fetal disposition of clinically important therapeutics and other xenobiotics. Admittedly, samples from pregnancies with documented mismanagement of blood glucose were unavailable, so it remains unknown if placental transporter expression is normal in T1DM-I and GDM-I because of proper management or because the human and rat placental response differs. Second, this study provides evidence that interindividual variability in placental transporter expression is comparatively less than that observed in other tissues. This suggests that disease-induced transporter expression changes may be easier to detect in the placenta, as seen in the recent work by Mason et al [57]. Future studies investigating the role of maternal disease in determining placental transporter expression will allow for a better understanding of the disposition of drugs and other xenobiotics during diseased-states in pregnancy.

## Supporting Information

Data S1 **Tables describing key patient variables.** In accordance with the recommendations of Nelson and Burton, supplemental tables describing key patient variables were compiled for each of this study’s three groups.
(DOC)

Data S2 **Representative western blots.** Representative blots showing MRP2, MDR1, BCRP, and β-actin expression in samples from each of this study’s three groups.
(DOC)
