# Supplementary figures and images for: Expression of ABC Efflux Transporters in Placenta from Women with Insulin-Managed Diabetes
Source: PLoS One. 2012 Apr 27;7(4):e35027. doi: 10.1371/journal.pone.0035027 (PMC3338746; doi:10.1371/journal.pone.0035027)

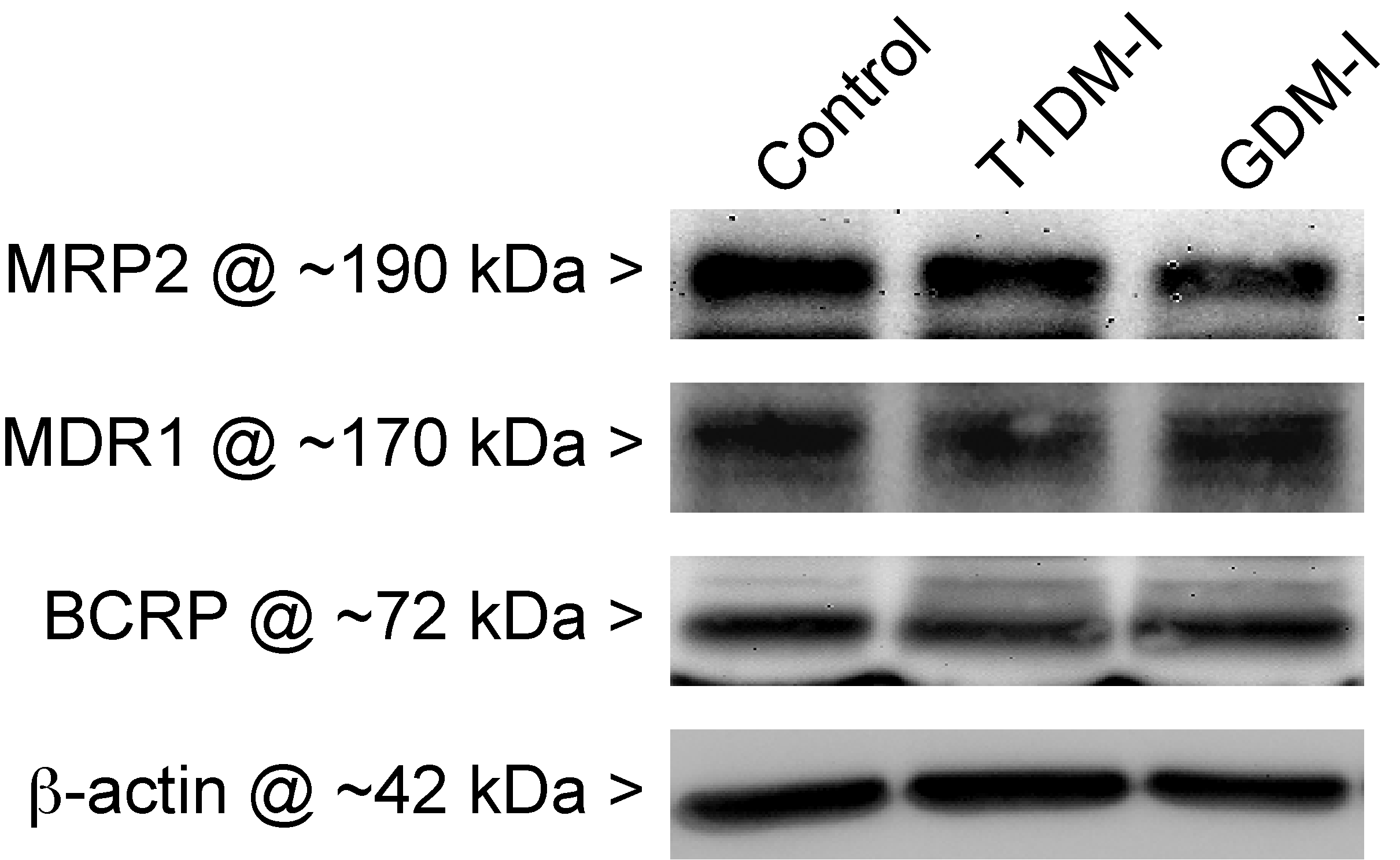

Supplement: Data S1 — Tables describing key patient variables. In accordance with the recommendations of Nelson and Burton, supplemental tables describing key patient variables were compiled for each of this study’s three groups. (DOC) [file pone.0035027.s001.doc]
