# Supplementary material for: Expression of ABC Efflux Transporters in Placenta from Women with Insulin-Managed Diabetes
Source: PLoS One. 2012 Apr 27;7(4):e35027. doi: 10.1371/journal.pone.0035027 (PMC3338746; doi:10.1371/journal.pone.0035027)
Supplement: Data S2 — Representative western blots. Representative blots showing MRP2, MDR1, BCRP, and β-actin expression in samples from each of this study’s three groups. (DOC) [file pone.0035027.s002.doc]

| **Table 1A – Term controls (n = 14)** | | | | |
| --- | --- | --- | --- | --- |
| **Parameter** | **Clinical Characteristics of Pregnancies for Placentas Studied** | | | |
| **Gravidity** | Median = 2 | 25-75% = 1-3 | Range = 1-4 |  |
| **Parity** | Median = 2 | 25-75% = 1-2 | Range = 1-4 |  |
| **Gestational age (weeks)** | Average = 39.3 | SD = 1 |  |  |
| **Maternal age**  **(years)** | Average = 32 | SD = 4 | Range = 25-43 |  |
| **Race** | Black = 0 | White = 11 | Asian = 3 | Other = 0 |
| **Ethnicity** |  |  |  |  |
| **Prenatal medications** | Iron: 1 | Other (list):  Acetaminophen: 1  Cefazolin: 1  Diclofenac: 1  Flonase: 1  Gravol: 1  H1N1 vaccine: 2  Maternal vitamins: 9  Tamiflu: 1 | None: 1 | Unknown: 1 |
| **Drugs** | Cigarettes,  n = 0 | Alcohol,  n = 1 | Other (list): | Unknown |
| **Previous prenatal admission(s)** | Yes,  n = 0 | No,  n = 0 | Diagnoses, if yes: | Unknown,  n = 14 |
| **Blood pressure**  **<140/90 mmHg** | Yes,  n = 14 | No,  n = 0 | Unknown,  n = 0 |  |
| **Screened for diabetes** | Yes,  n =14 | No,  n = 0 | Unknown,  n = 0 |  |
| **Antibiotics in labor** | None,  n = 6 | Penicillin,  n = 0 | Other (list):  n = 8 (C/S prophylaxis) | Unknown,  n = 0 |
| **Beta strep status** | Positive,  n = 0 | Negative,  n = 0 | Unknown,  n = 14 |  |
| **Antenatal steroids** | Yes,  n = 0 | No,  n = 14 | If yes, week GA | Unknown,  n = 0 |
| **Magnesium sulfate** | Yes,  n = 0 | No,  n = 0 | Unknown,  n =14 |  |
| **Anesthesia** | Epidural | Narcotics | General | Other/none |
| **Cervical ripening agent** | prostaglandin E_1_,  n = 0 | prostaglandin E_2_,  n = 2 | Mechanical | Other (list): |
| **Labor** | Yes/no  Yes: 6  No: 8 | If yes, hours:  01h 58m  04h 05m  04h 11m  05h 20m  07h 12m  17h 50m |  |  |
| **Delivery mode**  **(n = # patients)** | C-section,  Repeat,  no labor:  n = 6 | C-section,  Repeat,  with labor:  n = 1 | C-section, primary,  no labor:  n = 2 | C-section, primary,  with labor:  n = 0 |
| **Maternal oxygen given at delivery?** | Yes,  n = 0 | No,  n = 0 | Unknown,  n = 14 |  |
| **Birth weight**  **(grams)** | Average = 3481 | SD = 417 |  |  |
| **Placental weight**  **(grams)** | Average = 608 | SD = 127 | Unknown,  n = 0 |  |
| **Baby’s sex** | Female = 7 | Male = 7 | Unknown,  n = 0 |  |
| **Delivery to processing (mins)** | n = 1; 30 min | SD = n/a | Unknown,  n =13 |  |

| **Table 1B – Insulin-managed type 1 diabetes mellitus (n = 8)** | | | | |
| --- | --- | --- | --- | --- |
| **Parameter** | **Clinical Characteristics of Pregnancies for Placentas Studied** | | | |
| **Gravidity** | Median = 2 | 25-75% = 2-6 | Range = 1-7 |  |
| **Parity** | Median = 2 | 25-75% = 1-3 | Range = 1-4 |  |
| **Gestational age (weeks)** | Average = 38 | SD = 0.8 |  |  |
| **Maternal age**  **(years)** | Average = 33 | SD = 3 | Range = 27-37 |  |
| **Race** | Black = 0 | White = 6 | Asian = 1 | Other = 1 East Indian |
| **Ethnicity** |  |  |  |  |
| **Prenatal medications** | Iron: 1 | Other (list):  Acetaminophen: 1  ASA: 1  Diclectin: 2  Effexor: 1  Gravol: 1  Insulin: 8  Levothyroxine: 2  Maternal vitamins: 7  Metoclopramide: 1  RhIG: 1  Tums: 1  Vitamin D: 1  Zantac: 1 | None: 0 | Unknown: 0 |
| **Drugs** | Cigarettes,  n = 0 | Alcohol,  n = 0 | Other (list): | Unknown |
| **Previous prenatal admission(s)** | Yes,  n = 0 | No,  n = 0 | Diagnoses, if yes: | Unknown,  n = 8 |
| **Blood pressure**  **<140/90 mmHg** | Yes,  n = 8 | No,  n = 0 | Unknown,  n = 0 |  |
| **Screened for diabetes** | Yes,  n = 0 | No,  n = 8 (all with T1DM) | Unknown,  n = 0 |  |
| **Antibiotics in labor** | None,  n = 5 | Penicillin,  n = 0 | Other: (list)  n = 2 (C/S prophylaxis) | Unknown,  n = 1 |
| **Beta strep status** | Positive,  n = 0 | Negative,  n = 0 | Unknown,  n = 8 |  |
| **Antenatal steroids** | Yes,  n = 0 | No,  n = 8 | If yes, week GA | Unknown |
| **Magnesium sulfate** | Yes,  n = 1 | No,  n = 7 |  |  |
| **Anesthesia** | Epidural | Narcotics | General | Other/none |
| **Cervical ripening agent** | prostaglandin E_1_,  n = 0 | prostaglandin E_2_,  n = 1 | Mechanical | Other (list): |
| **Labor** | Yes/no  Yes = 5  No = 3 | If yes, hours:  05h 16m  08h 30m  08h 47m  08h 48m  21h 39m |  |  |
| **Delivery mode**  **(n = # patients)** | C-section,  Repeat,  no labor:  n =2 | C-section,  Repeat,  with labor:  n = 0 | C-section, primary,  no labor:  n = 1 | C-section, primary,  with labor:  n = 0 |
| **Maternal oxygen given at delivery?** | Yes,  n = 0 | No,  n = 0 | Unknown,  n = 8 |  |
| **Birth weight**  **(grams)** | Average = 3705 | SD = 542 |  |  |
| **Placental weight**  **(grams)** | Average = 666 | SD = 101 | Unknown,  n = 0 |  |
| **Baby’s sex** | Female = 2 | Male = 6 | Unknown,  n = 0 |  |
| **Delivery to processing (mins)** | Average = n/a | SD = n/a | Unknown,  n = 8 |  |

| **Table 1C – Insulin-managed gestational diabetes mellitus (n = 13)** | | | | |
| --- | --- | --- | --- | --- |
| **Parameter** | **Clinical Characteristics of Pregnancies for Placentas Studied** | | | |
| **Gravidity** | Median = 2 | 25-75% = 2-3 | Range = 1-4 |  |
| **Parity** | Median = 2 | 25-75% = 1-2 | Range = 1-3 |  |
| **Gestational age (weeks)** | Average = 38.8 | SD = 0.7 |  |  |
| **Maternal age**  **(years)** | Average = 35 | SD = 4 | Range = 28-43 |  |
| **Race** | Black = 1 | White = 9 | Asian = 1 | Other = 1 East Indian;  1 1/4 Asian, 3/4 White |
| **Ethnicity** |  |  |  |  |
| **Prenatal medications** | Iron: 0 | Other (list):  Acetaminophen: 1  Ca: 1  Colace: 1  Diclectin: 1  Diclofenac: 1  Insulin: 13  Maternal vitamins: 8  Mg: 1  Prometrium: 1  Seasonal influenza vaccine: 1  Sulfatrim: 1  Ventolin: 1 | None: 0 | Unknown: 0 |
| **Drugs** | Cigarettes,  n = 1 | Alcohol,  n = 0 | Other (list): | Unknown |
| **Previous prenatal admission(s)** | Yes,  n = 0 | No,  n = 0 | Diagnoses, if yes: | Unknown,  n = 13 |
| **Blood pressure**  **<140/90 mmHg** | Yes,  n = 12 | No,  n = 1 | Unknown |  |
| **Screened for diabetes** | Yes  n =13 | No  n = 0 | Unknown |  |
| **Antibiotics in labor** | None,  n = 0 | Penicillin,  n = 1 | Other (list):  n = 7, C/S prophylaxis; 1, Vancromycin | Unknown,  n = 0 |
| **Beta strep status** | Positive,  n = 1 | Negative,  n = 12 | Unknown,  n = 0 |  |
| **Antenatal steroids:** | Yes,  n = 1 | No,  n = 12 | If yes, week GA  = 2 courses of Celestone | Unknown |
| **Magnesium sulfate** | Yes,  n = 0 | No,  n = 13 |  |  |
| **Anesthesia** | Epidural | Narcotics | General | Other/none |
| **Cervical ripening agent** | prostaglandin E_1_ | prostaglandin E_2_ | Mechanical | Other (list): |
| **Labor** | Yes/no  Yes: 5  No: 8 | If yes, hours:  00h 02m  09h 05m  12h 03m  13h 01m  19h 16m |  |  |
| **Delivery mode**  **(n = # patients)** | C-section,  Repeat,  no labor:  n = 6 | C-section,  Repeat,  with labor:  n = 0 | C-section,  primary,  no labor:  n = 2 | C-section,  primary,  with labor:  n = 0 |
| **Maternal oxygen given at delivery?** | Yes,  n = 0 | No,  n = 0 | Unknown,  n = 13 |  |
| **Birth weight**  **(grams)** | Average = 3301 | SD = 404 |  |  |
| **Placental weight**  **(grams)** | Average = 661 | SD = 175 | Unknown,  n = 0 |  |
| **Baby’s sex** | Female = 8 | Male = 5 | Unknown,  n = 0 |  |
| **Delivery to processing (mins)** | Average = n/a | SD = n/a | Unknown,  n = 13 |  |
